# Expansion of the Metal‐Involving Noncovalent Interaction Repertoire: The Case of Pd(II) and Pt(II) Triel Bonding

**DOI:** 10.1002/cphc.70485

**Published:** 2026-07-07

**Authors:** Rosa M. Gomila, Antonio Frontera

**Affiliations:** ^1^ Department of Chemistry Universitat de les Illes Balears Palma de Mallorca Baleares Spain

**Keywords:** π-hole, DFT calculations, metals as nucleophiles, supramolecular chemistry, triel bonding

## Abstract

Supramolecular chemistry has traditionally focused on noncovalent interactions where p‐block elements act as electron acceptors via σ‐hole or π‐hole regions. While late transition metals in square planar geometries are typically viewed as electrophilic centers, recent evidence has shown that Pd and Pt complexes can act as nucleophiles through their filled d_z_2 orbitals. This behavior has been characterized for halogen, chalcogen, and pnictogen bonds, but triel bonding involving these metals remains unexplored. In this work, we analyze the interaction between square planar Pd and Pt model compounds and various triel bond donors, including BH_3_, BF_3_, BCl_3_, BBr_3_, and an aromatic boron derivative. Using DFT calculations, we evaluate the energetic and geometric features of these assemblies, which reveal the formation of moderately strong noncovalent bonds. The nature of these interactions is further characterized using the energy decomposition analysis (EDA), quantum theory of atoms in molecules (QTAIM), and natural bond orbital (NBO) analysis, confirming the significant charge transfer from the metal d_z_2 orbital to the triel acceptor. Additionally, a search of the Cambridge Structural Database (CSD) provides experimental validation for the existence of these interactions in the solid state. Our results establish the triel bond as a new member of the family of metal‐involving noncovalent interactions, offering new possibilities for crystal engineering and supramolecular design.

## Graphical abstract

This study reveals that square planar Pd and Pt complexes can act as nucleophiles, forming directional triel bonds with boron‐based acceptors. Using energy decomposition analysis (EDA), quantum theory of atoms in molecules (QTAIM), and natural bond orbital (NBO), we demonstrate a transition from dispersion‐driven to dative‐covalent interactions. The Cambridge Structural Database (CSD) provides experimental validation, establishing these contacts as a new tool for supramolecular design.

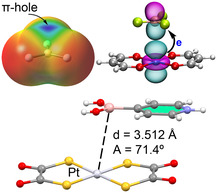

## 1 Introduction

Supramolecular chemistry has emerged as a cornerstone of modern chemical science [1, 2], focusing on the assembly of molecular architectures through noncovalent interactions [3]. These forces, ranging from hydrogen bonding [4] to van der Waals interactions [5], govern the formation of complex systems in biological processes, materials science, and catalysis. Unlike covalent bonds, noncovalent interactions provide the flexibility and reversibility necessary for dynamic molecular recognition and self‐assembly, allowing for the precise design of functional supramolecular materials with tailored properties [6, 12].

Among the various noncovalent forces, σ–hole and π–hole interactions have gained significant attention due to their highly directional nature and tunable strength [13, 14]. These interactions occur when an electron‐deficient region (the hole) on an atom or a molecular fragment interacts with a nucleophilic site [15]. While halogen bonding [16, 17] is perhaps the most well‐documented example, recent years have seen a surge in interest regarding other p‐block elements [18, 20]. These interactions are characterized by the electrostatic attraction between the positive potential of the σ–hole or π–hole and a Lewis base, and they play a vital role in crystal engineering [21] and the stabilization of reactive intermediates [22].

The expansion of noncovalent bonding nomenclature has sparked debate within the chemical community regarding the most appropriate framework for classifying these interactions. One approach, endorsed by recent IUPAC recommendations, adopts a systematic taxonomy that categorizes interactions according to the periodic group of the electrophilic atom, giving rise to terms such as halogen [17], chalcogen [19], and pnictogen bonding [20], thereby emphasizing the distinct chemical character of each interacting element [23]. An alternative, physics‐based nomenclature focuses instead on the properties of the electron‐depleted region itself, employing generalized terms such as σ–hole, π–hole, or p–hole bonds to underscore the mechanistic similarities shared across different groups [24]. Beyond the question of naming, the conventional designation of these forces as strictly noncovalent has also been called into question: advanced electronic structure analyses frequently reveal significant charge transfer and orbital mixing that approach the character of dative covalent coordination, and it has been argued that the term “noncovalent” is fundamentally a misnomer for interactions rooted in the same orbital‐level covalency that governs chemical bonding more broadly [25].

Specifically, the triel bond, an interaction involving elements of group 13 (B, Al, Ga, In, Tl) as electron acceptors, represents a unique category of noncovalent bonding [26, 31]. Triel atoms often possess a vacant p‐orbital or a region of low electron density (π‐hole) perpendicular to the molecular plane in planar species like BX_3_. This allows them to engage in significant interactions with various electron donors [32]. Although triel bonds are known to be particularly strong and have been explored in the context of molecular recognition and catalysis, their investigation remains less exhaustive compared to their halogen, chalcogen, or pnictogen counterparts.

**SCHEME 1:**
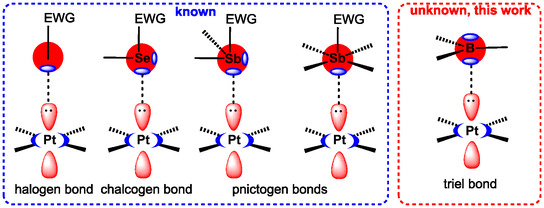
Left: previously reported σ–hole interactions involving d_z_2 metal donors. Right: π–hole triel bonding interactions reported in this work.

A fascinating development in this field is the recognition of late transition metals with low coordination numbers, such as square planar Pd and Pt complexes, acting as nucleophiles [33]. These metals possess a doubly occupied d_z_2 orbital that is energetically accessible and spatially oriented to act as an electron donor [34]. Recent studies (see Scheme 1) have demonstrated that these metal centers can effectively interact with σ‐holes in halogen [35], chalcogen [36, 37], and pnictogen bonding [38, 39]. In these cases, the metal center functions as the Lewis base, donating electron density from the d_z_2 orbital into the antibonding σ* or vacant p‐orbitals of the acceptor atom, thereby directing the supramolecular assembly.

**SCHEME 2:**
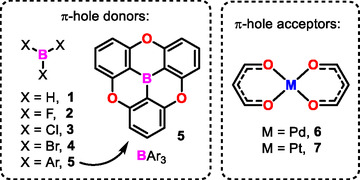
Chemical drawings of the π hole donors **1–** **5** and acceptors **6** and **7** used in this work.

Despite the progress in metal‐involving noncovalent interactions, the participation of square planar metal complexes in triel bonding has remained largely unexplored. In this manuscript, we provide a comprehensive analysis of the energetic and geometric features of the interaction between Pd and Pt model complexes and various triel donors, including BH_3_, BF_3_, BCl_3_, BBr_3_, and an aromatic B derivative (see Scheme 2). We characterize these moderately strong supramolecular assemblies using the energy decomposition analysis (EDA), quantum theory of atoms in molecules (QTAIM), and natural bond orbital (NBO) analysis to provide a deep understanding of the electrostatic, dispersive, and covalent contributions to the interaction. Furthermore, we provide experimental evidence for the existence of this bonding mode through an analysis of the CSD crystal structure database, establishing the triel bond as a significant tool in metal‐mediated crystal engineering.

## 2 Theoretical Methods

All geometry optimizations were performed using the Gaussian 16 software package [40]. The geometries of all compounds and supramolecular complexes were fully optimized without symmetry constraints. The PBE0 hybrid functional [41] was employed in combination with Grimme’s D3 dispersion correction to accurately account for long‐range dispersion effects, which are critical in describing noncovalent interactions [42]. The def2‐TZVP basis set was utilized for all atoms to ensure a high‐quality description of the electronic density and the polarizability of the heavier halogen atoms and metal centers [43]. For heavy atoms, including Pd and Pt, the def2‐TZVP basis set incorporates effective core potentials (ECPs) that implicitly account for scalar relativistic effects, which are non‐negligible for elements of this atomic weight and are essential for an accurate description of their electronic structure and bonding [43].

The molecular electrostatic potential (MEP) surfaces were computed at the PBE0‐D3/def2‐TZVP level of theory to quantify the electrophilic π‐holes on the boron derivatives and the nucleophilic metal centers. The resulting MEP maps and potential extrema (*V*_s,max_) were visualized and plotted using the GaussView 6.0 interface.

The topological properties of the electron density were analyzed through the atoms in molecules (AIM) theory using the AIMAll program [44]. This analysis was performed to confirm the existence of the noncovalent bonds and to characterize the nature of the triel bonding interactions via the identification of bond critical points (BCPs) and the evaluation of the electron density (*ρ*) at these points. EDA was performed using an improved version of the Kitaura–Morokuma method [45] as implemented in the Turbomole 7.8 program [46]. NBO [47] analysis was performed using the NBO7 program [48] and plotted using the VMD software [49].

## 3 Results and Discussion

### 3.1 Preliminary MEP Analysis of Compounds 1–7

**FIGURE 1:**
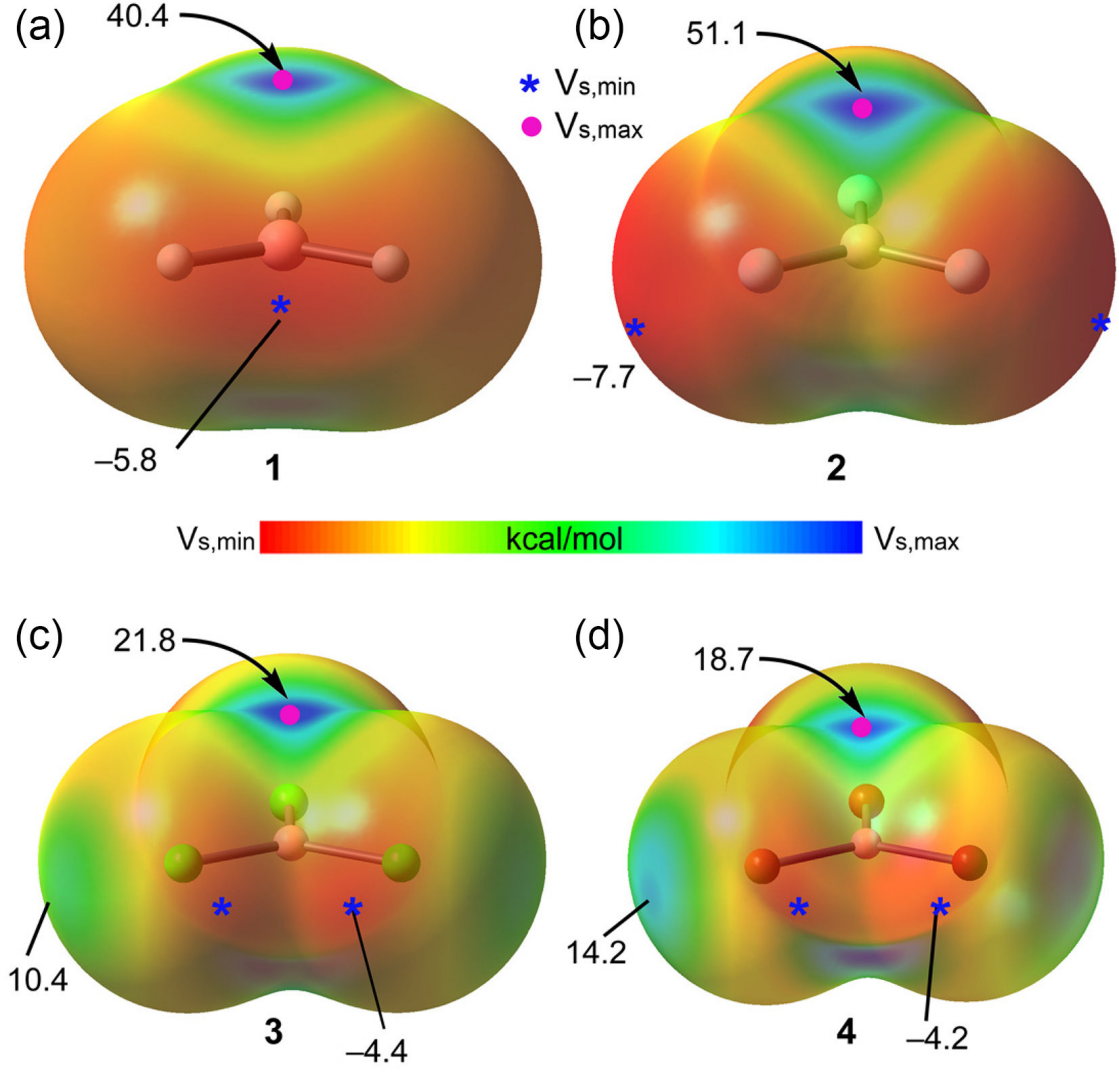
MEP surfaces of the triel donors BH_3_ (a), BF_3_ (b), BCl_3_ (c), and BBr_3_ (d) calculated at the 0.001 au isosurface. The positions and values of the MEP maxima (π‐holes) and minima are indicated in kcal/mol. For BCl_3_ and BBr_3_, the σ‐holes located on the halogen atoms are also indicated.

We began our study by analyzing the MEP surfaces of the selected triel bond donors to evaluate their propensity to interact with the metal nucleophiles. The MEP surfaces for BH_3_, BF_3_, BCl_3_, and BBr_3_ are illustrated in Figure 1. In all instances, a well‐defined region of positive potential, or π‐hole, is observed centered at the B‐atom, as is characteristic for these planar species. The intensity of the π‐hole follows the trend BF_3_ > BH_3_ > BCl_3_ > BBr_3_. The maximum value is found for BF_3_ (51.1 kcal/mol), which can be attributed to the strong inductive electron‐withdrawing effect of the fluorine atoms combined with their low basicity. This combination effectively minimizes the LP(F) → p_z_(B) back‐donation, leaving the boron center significantly electron‐deficient. The corresponding MEP minimum for BF_3_ is located at the F‐atoms (–7.7 kcal·mol^–1^). Interestingly, BH_3_ also exhibits a remarkably large π‐hole value (40.4 kcal·mol^–1^), with the MEP minimum situated in the molecular plane between the H‐atoms (–5.8 kcal·mol^–1^). In contrast, the π‐hole values for BCl_3_ and BBr_3_ are significantly lower (21.8 and 18.7 kcal·mol^–1^, respectively). This reduction is likely due to the decreased electronegativity of Cl and Br compared to F, alongside a more effective lone pair (LP) → p_z_(B) orbital donation that partially neutralizes the positive potential at the boron center. Notably, for the heavier halogen derivatives, the halogen atoms themselves exhibit sigma‐holes along the extension of the B─X bonds, with values of 10.4 kcal·mol^–1^ for Cl and 14.2 kcal·mol^–1^ for Br. The MEP minima for these molecules are associated with the halogen LP regions (–4.4 kcal·mol^–1^ for Cl and –4.2 kcal·mol^–1^ for Br). This dual electrophilic nature (π‐hole at B and σ‐hole at X) highlights the complex potential energy surfaces of these triel donors.

**FIGURE 2:**
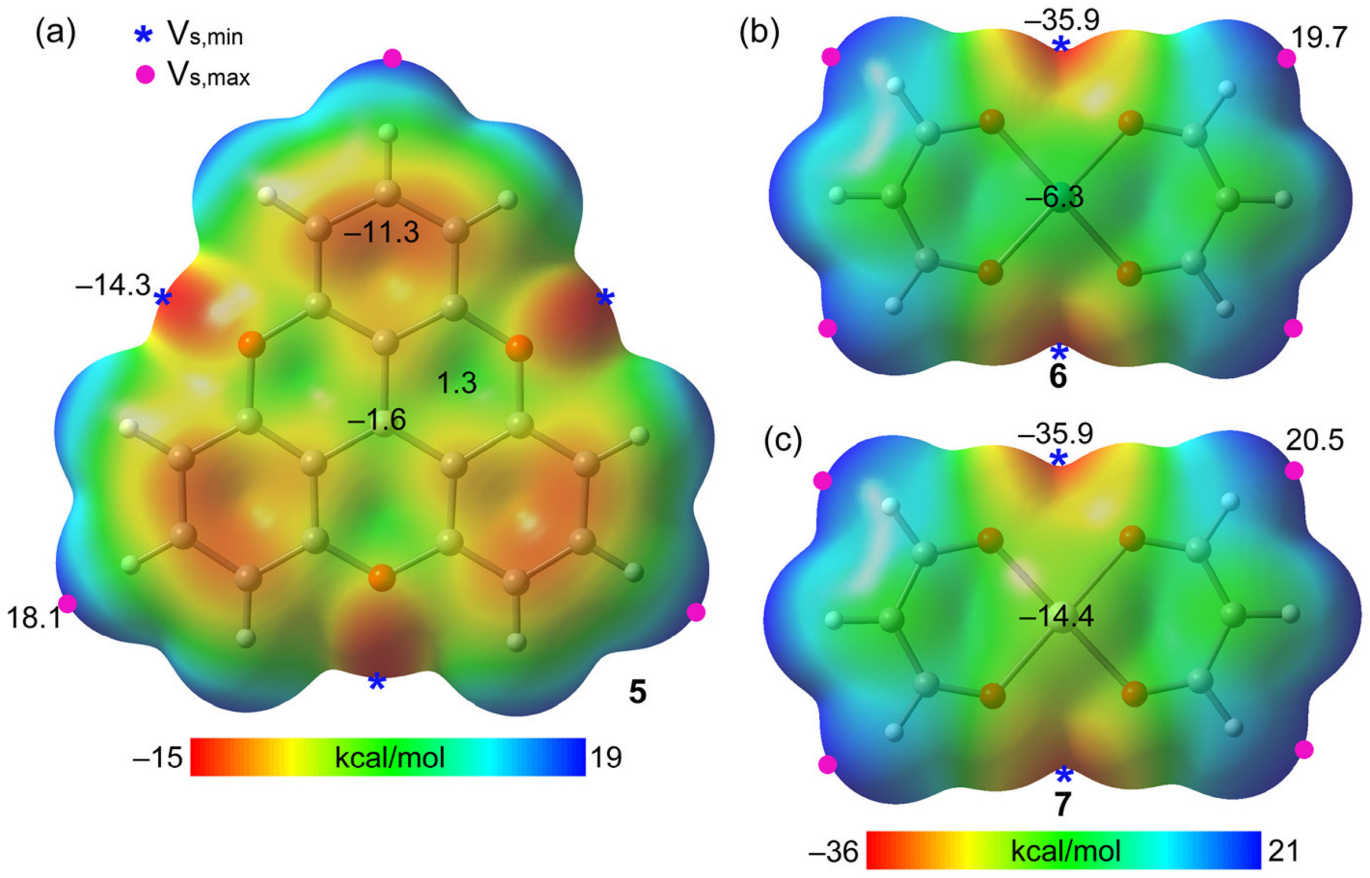
MEP surfaces of the aromatic boron derivative **5** (a), the Pd(II) complex **6** (b), and the Pt(II) complex **7** (c) calculated at the 0.001 au isosurface. The values of the MEP maxima and minima are indicated in kcal·mol^–1^. The negative MEP values at the metal centers (Pd and Pt) highlight their nucleophilic character.

The MEP surface of the aromatic boron derivative 5 (Figure 2a) reveals a distinct electronic distribution compared to the simple triel donors. The MEP maximum is located at the aromatic H‐atoms (18.1 kcal·mol^–1^), while the MEP minima are found at the bridging O‐atoms (–14.3 kcal·mol^–1^). Notably, the MEP value at the B‐atom is slightly negative (–1.6 kcal·mol^–1^). This unexpected result suggests that the π → p_z_(B) electron donation from the adjacent oxygen atoms and the aromatic system is sufficient to compensate for the Lewis acidity typically expected for boron derivatives. Furthermore, the MEP is negative over the C_6_ aromatic rings (–11.3 kcal·mol^–1^) and slightly positive over the 1,4‐oxaborinine rings (1.3 kcal/mol).

Regarding the metal complexes **6** and **7** (Figure 2b,c), the MEP surfaces show that the global minima are located in the molecular plane at the O‐atoms of the ligands (–35.9 kcal·mol^–1^). The maxima are found at the ligand H‐atoms, with values of 19.7 kcal·mol^–1^ for the Pd complex **6** and 20.5 kcal/mol for the Pt complex **7**. Crucially, the MEP analysis confirms the nucleophilicity of the metal centers. The MEP values at the metal positions are −6.3 kcal/mol for Pd and −14.4 kcal/mol for Pt, supporting the availability of the d_z_2 LP for noncovalent interactions. This higher nucleophilicity of the Pt center compared to Pd is consistent with the greater relativistic expansion of the 5d orbitals.

### 3.2 Energetic Analysis

**SCHEME 3:**
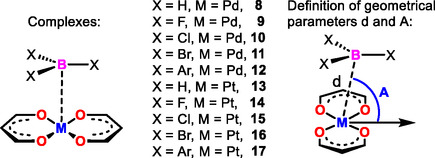
Chemical drawings of complexes **8–** **17** and the definition of geometric parameters d and A gathered in Table 1.

The interaction energies (*E*_int_) and equilibrium distances (*d*) for the complexes (see Scheme 3) formed between the metal nucleophiles (**6** and **7**) and the triel donors (BH_3_, BF_3_, BCl_3_, BBr_3_, and the aromatic derivative **5**) are summarized in Table 1. In all cases, the Pt complexes exhibit significantly stronger binding energies compared to their Pd counterparts. For instance, the interaction of Pt complex **7** with BH_3_ (complex **13**, *E*_int_ = –17.8  kcal·mol^–1^) is considerably stronger than the corresponding Pd complex **8** (*E*_int_ = –10.2 kcal·mol^–1^). This trend is in excellent agreement with the MEP analysis, which showed a more negative potential at the Pt center (–14.4 kcal·mol^–1^) compared to Pd (–6.3 kcal·mol^–1^), confirming the higher nucleophilicity of the heavier metal.

**TABLE 1:** Interaction energies (*E*_int_, kcal·mol^–1^), M···B interaction distances (*d*, Å) and angles (*A*, °), see Scheme 3 for definition of *d* and *A*. The density at the BCP characterizing the triel bond is indicated (ρ, a.u.).

Complex	E_int_	*d*	*A*	*ρ* a
**8**	−10.2	2.453	89.2	0.0362
**9**	−4.5	3.179	76.4	0.0085
**10**	−6.5	3.379	74.9	0.0075
**11**	−7.6	3.423	74.3	0.0074
**12**	−12.6	3.365	86.8	0.0079
**13**	−17.8	2.362	89.2	0.0515
**14**	−6.6	3.003	87.9	0.0154
**15**	−7.3	3.316	81.3	0.0101
**16**	−8.3	3.365	78.9	0.0098
**17**	−14.0	3.293	86.6	0.0115

a In complexes **12** and **17**, the BCP connects the metal to the C‐atom.

The interaction with the aromatic triel donor **5** (complexes **12** and **17**) yields the second largest binding energies in the series (–12.6 and –14.0 kcal·mol^–1^ for Pd and Pt, respectively) after complex **13**. This enhanced stability is likely attributed to the large aromatic surface of the donor, which allows for secondary dispersive interactions or π‐stacking contributions with the square planar metal complexes in addition to the triel bond. Furthermore, the exceptionally short distance observed for complexes **8** and **13** (2.453 and 2.362 Å, respectively) suggests a potential degree of covalent character, which will be further scrutinized.

A particularly intriguing result is the trend observed for the BX_3_ series (X = F, Cl, Br). While the π‐hole intensity follows the order BF_3_ > BCl_3_ > BBr_3_, the interaction energies follow the opposite trend. Specifically, for the Pt series, the binding energy increases from BF_3_ (complex **14**, –6.6 kcal·mol^–1^) to BCl_3_ (complex **15**, –7.3 kcal·mol^–1^) and BBr_3_ (complex **16**, –8.3 kcal/mol). This inverse relationship suggests that the electrostatic component (π‐hole magnitude) is not the sole governing factor in these triel bonds. The increase in strength with the heavier halogens likely points toward a significant contribution from dispersion forces and polarizability, or a more favorable orbital overlap between the metal d_z_2 and the boron p_z_ orbital as the halogens become less electronegative. This counterintuitive behavior will be analyzed in more depth in the following sections using QTAIM, EDA, and NBO analyses.

The analysis of the geometric parameters in Table 1 confirms the highly directional nature of the triel bond. The distance *d* varies significantly depending on the metal and the triel donor, being consistently shorter for the platinum complexes compared to the palladium counterparts. This trend is consistent with the stronger *E*_int_ values observed for Pt and reinforces the conclusion that the heavier metal acts as a more effective nucleophile. For instance, the distance d in the Pt···BH_3_ complex **13** (2.362 Å) is notably shorter than in its Pd counterpart **8** (2.453 Å); these two complexes are particularly representative as no secondary interactions are present to influence the geometry.

In the BX_3_ series (X = F, Cl, Br), the distance d increases as the halogen becomes heavier and more polarizable. For the Pt complexes, the distance d ranges from 3.003 Å in the BF_3_ complex (**14**) to 3.365 Å in the BBr_3_ complex (**16**), while for the Pd series, it increases from 3.179 Å (**9**) to 3.423 Å (**11**). This increase in the equilibrium distance perfectly mirrors the trend in the MEP values, where the π‐hole becomes less positive from F to Br. This correlation suggests that the triel bond distance is highly sensitive to the Lewis acidity of the boron center as defined by its electrostatic potential.

The directionality of the interaction is described by the angle A, measured between the B atom, the metal center, and the bisector of the O─M─O bond (see Scheme 3, right). In all cases, A values remain close to 90°, indicating that the boron atom approaches the metal center almost perpendicularly to the square coordination plane. Notably, the A values for the Pt complexes are generally closer to 90° than those of the Pd counterparts. This superior directionality in the Pt series is in line with their stronger interaction energies and shorter equilibrium distances, reflecting a more effective and specialized orbital overlap. Such a geometry is ideal for maximizing the overlap between the vacant p_z_ orbital of the boron (the π‐hole) and the filled d_z_2 orbital of the Pd or Pt center. The maintenance of this nearly orthogonal approach across the diverse set of donors highlights the stereoelectronic control exerted by the metal d_z_2 LP in directing these supramolecular assemblies.

**FIGURE 3:**
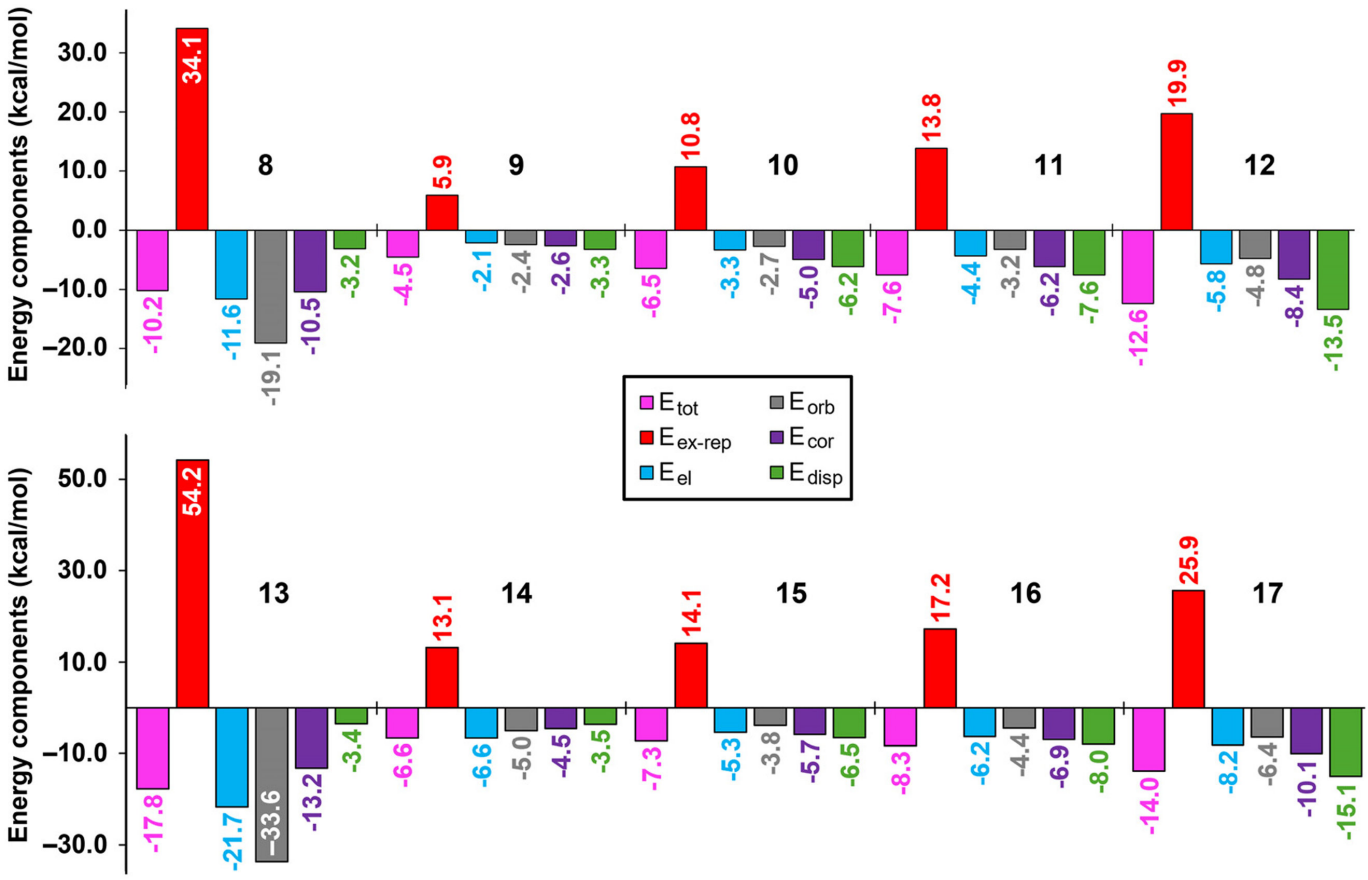
Energy decomposition analysis for complexes **8–** **17**.

To further differentiate the nature of the triel bonds across the different families of complexes, we performed an EDA. The results, summarized in Figure 3, provide a clear rationale for the energetic trends and the distinct behavior of the BH_3_, BX_3_, and BA_r3_ series. For the BH_3_ complexes (**8** and **13**), the EDA profile is markedly different from the remaining systems, a difference that can be rationalized on the basis of both structural and electronic factors. Unlike the halogenated BX_3_ derivatives, BH_3_ bears no substituents capable of engaging in lone‐pair‐to‐boron p‐orbital back‐donation. In BX_3_ systems, this internal electron donation partially saturates the vacant p orbital of boron, attenuating its Lewis acidity and limiting the depth of interaction with the metal fragment. In BH_3_, by contrast, the vacant p orbital remains fully exposed, resulting in significantly shorter equilibrium contact distances, nearly 1 Å shorter than those found in the halogenated analogs. At such short separations, both the electrostatic (*E*_el_, blue bars) and orbital (*E*_orb_, gray bars) attractive components are substantially enhanced. Notably, the *E*_orb_ term is the dominant contributor to the attraction (–19.1 and –33.6 kcal·mol^−1^ for **8** and **13**, respectively), indicative of a partial covalent character and reflecting efficient overlap between the metal d orbitals and the unshielded boron p orbital. However, these strong attractive forces are largely counterbalanced by a massive exchange‐repulsion component, which reduces the total interaction energy considerably.

In the halogenated BX_3_ series, all energy components are significantly smaller compared to the BH_3_ complexes. A key differentiating feature arises when comparing the Pd and Pt complexes within this group. In the Pd complexes (**9**‐**11**), the largest attractive term is dispersion (*E*_disp_, pink bars, up to –7.6 kcal·mol^–1^ in **11**), followed by the correlation term (*E*_cor_, dark violet bars, up to –6.2 kcal·mol^–1^ in **11**). *E*_disp_ and *E*_cor_ terms are particularly significant for the BCl_3_ and BBr_3_ complexes, aligning with the larger interaction energies observed for the heavier halogens despite their weaker π‐holes. In contrast, for the Pt complexes (**14**‐**16**), the electrostatic term becomes more important (ranging from –5.3 to –6.6 kcal·mol^–1^), which aligns with the MEP results showing Pt as a superior nucleophile. Indeed, electrostatics is the dominant attractive term in the Pt···BF_3_ complex (**14**). Overall, all individual components are larger for Pt than for Pd due to the shorter equilibrium distances.

Regarding the aromatic BAr_3_ complexes (**12** and **17**), the interaction is clearly dominated by the dispersion term (–13.5 and –15.1 kcal·mol^–1^, respectively), as is typical for systems governed by π‐stacking interactions and aligned with the large aromatic surface of compound **5**. This is followed by the *E*_cor_ and *E*_el_ contributions. The exchange‐repulsion term in these systems is larger than in the BX_3_ complexes, reflecting the increased steric demand and larger size of the triel donor molecules. It should be emphasized that the large binding affinities observed in these complexes are a direct consequence of the multicentered nature of the assembly. While the NBO analysis clearly demonstrates the existence of a genuine, directional triel bond, the parallel orientation of the square planar metal framework relative to the extended polycyclic aromatic surface of the triel donor allows the system to maximize van der Waals contacts across the entire molecular interface. This interpretation is strongly supported by the QTAIM topology, which reveals a dense network of nine separate BCPs interconnecting the two monomers. Consequently, the dispersion term acts as a massive global stabilizer owing to the large polarizable surface area, whereas the localized metal to boron orbital overlap serves as the directional anchor that dictates the specific supramolecular orientation. This synergistic interplay perfectly rationalizes why the system exhibits a dispersion‐dominated energy decomposition profile despite possessing a clear and robust dative character at the core bonding site.

### 3.3 QTAIM Analysis

**FIGURE 4:**
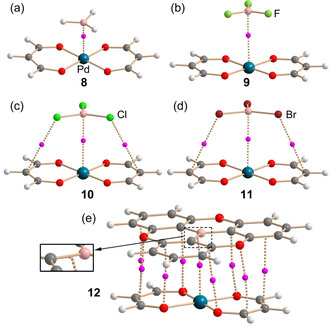
QTAIM distribution of bond critical points (pink spheres) and bond paths (dashed lines) for the Pd(II) complexes **8** (a), **9** (b), **10** (c), **11** (d), and **12** (e). The analysis confirms the primary triel bond and identifies auxiliary halogen‐ligand and π–π interactions.

To further characterize the nature of the interactions and complement the EDA analysis, we performed a topological analysis of the electron density using the QTAIM. Figure 4 illustrates the distribution of BCPs and bond paths for the Pd complexes (**8–** **12**). Since the topological features of the Pt series are identical to those of the Pd series, only the latter are shown to avoid repetition.

For the complexes involving BH_3_ (**8**, Figure 4a) and BF_3_ (**9**, Figure 4b), a single BCP and bond path interconnect the metal center and the boron atom. This confirms that a unique triel bond is the primary interaction responsible for the stabilization of these assemblies. The lower interaction energy observed for the BF_3_ complex compared to BH3 may be partially attributed to the spatial arrangement, where the electronegative F atoms are positioned over the negatively charged O‐atoms of the metal ligands, leading to a degree of electrostatic repulsion that offsets the π‐hole attraction.

In the case of BCl_3_ (**10**) and BBr_3_ (**11**) complexes (Figure 4c,d), the topological analysis reveals a more complex bonding pattern. In addition to the primary triel bond, extra BCPs and bond paths connect two halogen atoms of the triel donor to the central C‐atoms of the ligands. These auxiliary contacts, where the positive sigma‐holes of the Cl or Br atoms (identified in the MEP analysis) interact with the π‐system of the ligands, likely explain the larger interaction energies observed for BCl_3_ and BBr_3_ compared to BF_3_. This supplemental stabilization occurs despite the weaker π‐hole at the boron atom in the heavier triel donors and provides a physical manifestation of the large dispersion and correlation terms identified by the EDA.

For complex **12** (Figure 4e), the topology is significantly different. The primary bond path connects the metal atom to one of the C‐atoms of the aromatic ring rather than the boron atom directly. Furthermore, eight additional BCPs and bond paths interconnect the two monomers. This extensive network of interactions highlights the significant participation of the π‐systems of both molecules in the binding mechanism. These findings justify the exceptionally large dimerization energies observed for complexes **12** and **17**, indicating that while a triel‐type interaction is present, the assembly is strongly reinforced by π‐stacking contributions and perfectly justifies the dispersion‐dominated profile seen in the energy decomposition.

The topological properties of the electron density at the BCPs provide further quantitative evidence for the nature and strength of the triel bonds. The electron density values (*ρ*) at the BCPs for complexes **8**–**17** are listed in Table 1. The most striking feature of the QTAIM data is the exceptionally high ρ values for the BH_3_ complexes. Specifically, for the Pt···BH_3_ complex **13**, the electron density reaches 0.0515 au. This value is significantly higher than what is typically observed for purely noncovalent interactions and, as suggested by the remarkably short *d* value of 2.362 Å and significant orbital contribution discussed in the EDA section, confirms a degree of covalent character in this triel bond. This indicates a robust coordination that borders on a dative covalent bond. The Pd···BH_3_ counterpart (**8**) also shows a relatively high value of 0.0362 au, reinforcing the idea that BH_3_ is a particularly potent triel bond acceptor for these metal nucleophiles.

For the halogenated BX_3_ series and the aromatic derivative, the*ρ* values are lower, reflecting a more typical supramolecular character. Within the Pt series, the values range from 0.0154 a.u. for BF_3_ (**14**) to 0.0098 au for BBr_3_ (**16**). The value for BF_3_ (**14**) is notably higher than those of the heavier halogenated complexes (**15** and **16**), which is consistent with the shorter distance d observed for the fluorine derivative. In the Pd series, the *ρ* values remain very similar across the halogenated donors, ranging from 0.0074 to 0.0085 au. These values reflect the moderately strong nature of the triel bonds in these assemblies.

### 3.4 NBO Analysis

**FIGURE 5:**
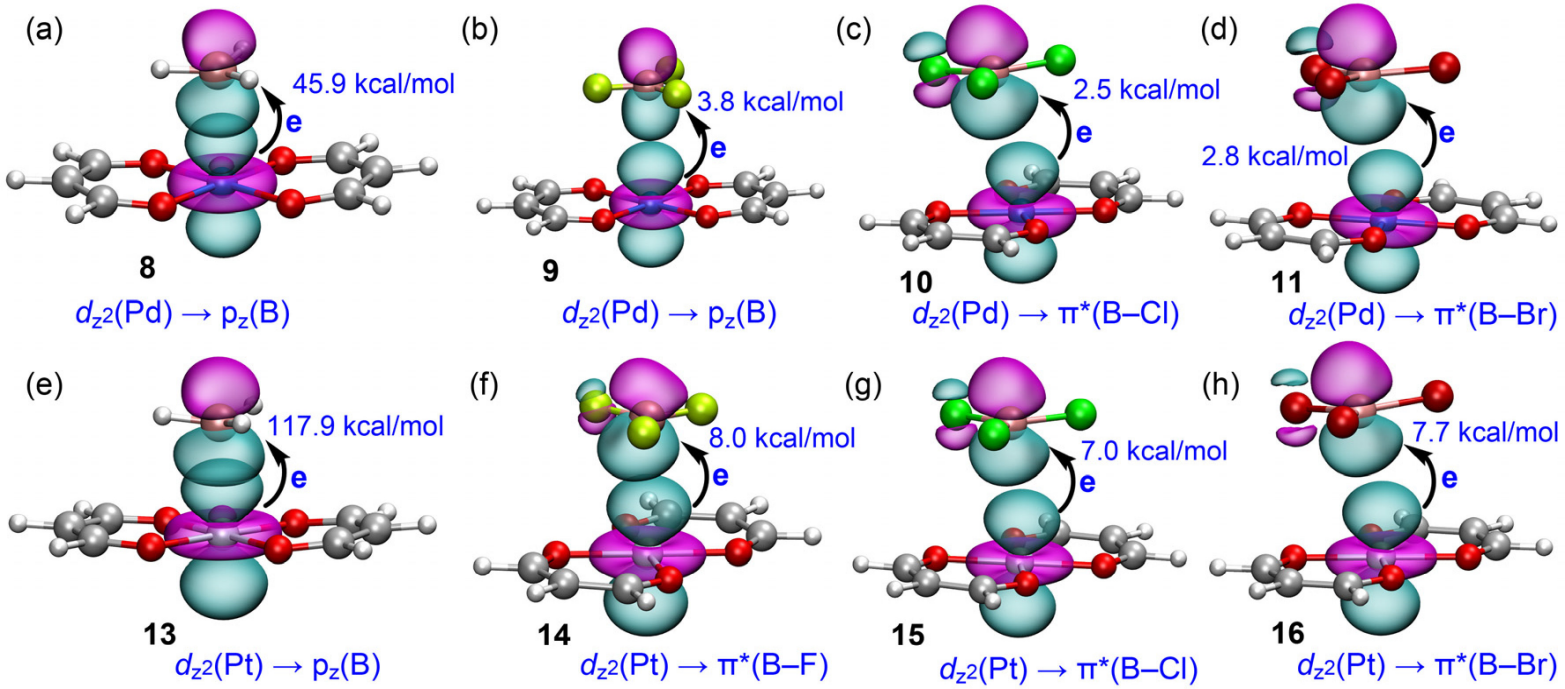
NBO plots showing the overlap between the filled metal d_z_2 orbital (donor) and the vacant p_z_ or π* B–X orbital (acceptor) for Pd complexes **8–** **11** (top, (a), (b), (c) and (d)) and Pt complexes **13–** **16** (bottom, (e), (f), (g) and (h)). The second‐order perturbation energies *E* ^(2)^ (kcal/mol) and the orbital plots are indicated.

To gain deeper insight into the nature of the triel bond and the charge transfer processes involved, we performed a NBO analysis. Figure 5 displays the donor–acceptor orbitals involved in the interaction for the BH_3_ and BX_3_ complexes of Pd (**8–** **11**) and Pt (**13–** **16**). The results reveal a significant disparity between the BH_3_ complexes and the halogenated BX_3_ derivatives.

For the complexes involving BH_3_, we observe exceptionally large second‐order perturbation energies *E* ^(2)^ associated with the d_z_2(M) → p_z_(B) electron donation. This is particularly evident in the overlap plots for complexes **8** and **13**, where the filled d_z_2 orbital of the metal and the vacant p_z_ orbital of the boron atom show a high degree of spatial complementarity. In the case of the Pt complex **13**, the interaction is strong enough to induce a slight pyramidalization of the BH_3_ moiety, which, combined with the short triel bond distance and high *E* ^(2)^ values, confirms a non‐negligible covalent character.

In contrast, for the halogenated series (X = F, Cl, Br), the *E* ^(2)^ energies range from 2.5 to 8.0 kcal·mol^–1^, which is typical for standard noncovalent bonding. Consistent with our previous energetic and geometric findings, the *E* ^(2)^ values are larger for the Pt complexes than for the Pd counterparts. This is attributed to the shorter interatomic distances and the larger, more diffuse nature of the Pt d_z_2 orbital, which facilitates better overlap with the acceptor.

An interesting stereoelectronic feature revealed by the NBO analysis is that for complexes **10**, **11**, and **14–** **16**, the electron density is not donated directly into a pure p_z_ atomic orbital of boron. Instead, the metal d_z_2 orbital interacts with a π‐antibonding B–X orbital (π* B–X). However, the orbital composition analysis shows that the p_z_ atomic orbital of boron remains the dominant contributor to this antibonding MO, with a weight exceeding 85%. This confirms that despite the involvement of B─X bonds in the acceptor orbital, the interaction remains fundamentally a triel bond centered on the boron π‐hole.

**FIGURE 6:**
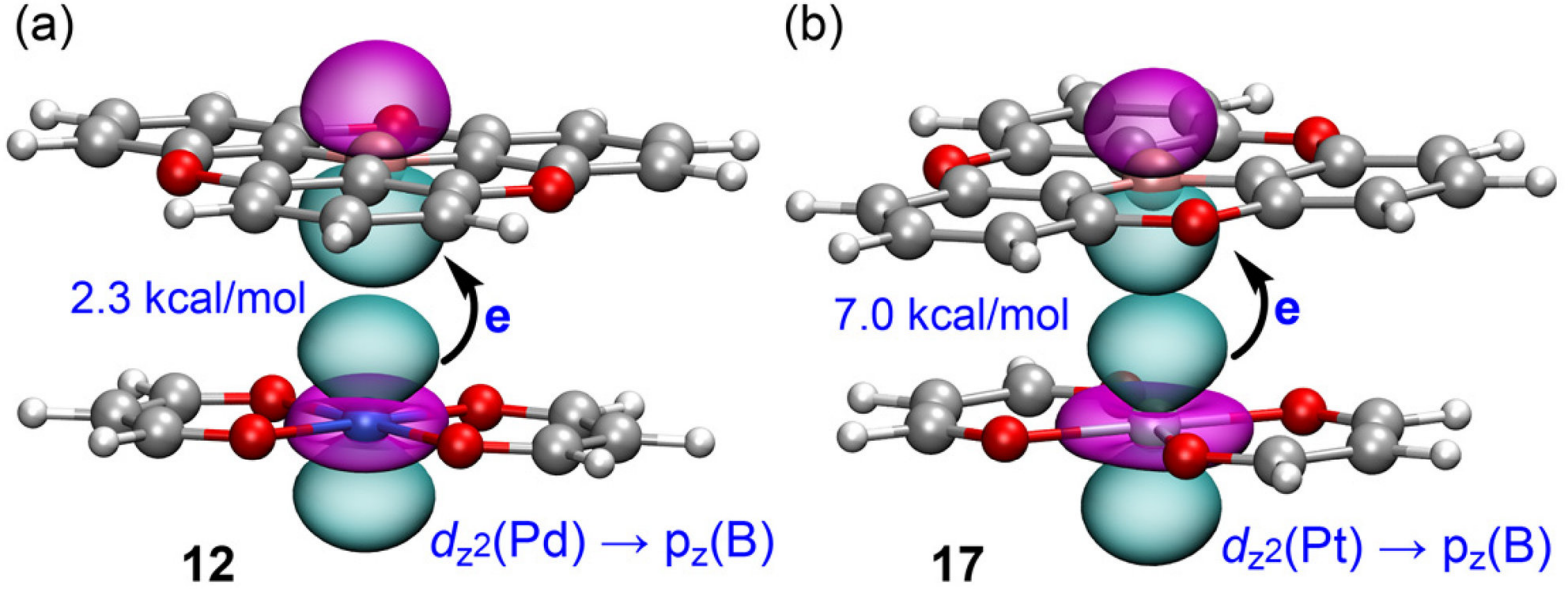
NBO surfaces representing the orbital interaction between the filled metal d_z_2 orbital (donor) and the vacant p_z_ orbital of boron (acceptor) for the aromatic BAr_3_ complexes **12** (a) and **17** (b). The *E* ^(2)^ energies and orbital overlaps confirm the presence of a triel bond contact.

The NBO analysis of the aromatic BAr_3_ complexes **12** and **17** (Figure 6) provides crucial evidence regarding the true nature of their interaction. While the QTAIM analysis previously showed a bond path connecting the metal center to a C‐atom of the aromatic ring rather than the boron atom, the NBO donor–acceptor results clearly show that a significant orbital interaction exists between the filled d_z_2 orbital of the metal and the vacant p_z_ orbital of the boron atom. This orbital overlap confirms that a triel bond contact is indeed present and contributes to the overall stability of the supramolecular assembly.

Consistent with the trends observed throughout the series, the Pt complex **17** exhibits a larger *E* ^(2)^ value compared to the Pd complex **12**. Interestingly, the magnitude of these *E* ^(2)^ values is comparable to those obtained for the BCl_3_ and BBr_3_ complexes. This similarity suggests that while the large aromatic system induces a different topological distribution of electron density and bond paths, the local metal‐to‐boron orbital communication remains a fundamental component of the binding mechanism. These results highlight that the NBO analysis is an essential tool in this case to unveil the triel bond character that might be otherwise obscured by the pervasive π‐stacking and dispersive forces indicated by the QTAIM bond paths.

Finally, it is informative to compare the *E* ^(2)^ values with the orbital components (*E*_orb_) derived from the EDA. For the BH_3_ complexes, both methods yield the highest values in the series, though the NBO values are significantly larger in magnitude. For the Pt complex **13**, the NBO *E* ^(2)^ is 117.9 kcal·mol^–1^ compared to an *E*_orb_ of –33.6 kcal·mol^–1^, while for the Pd complex **8**, the NBO *E* ^(2)^ is 45.9 kcal/mol compared to an *E*_orb_ of –19.1 kcal/mol. In the halogenated BX_3_ series, the values are more modest; for the BCl_3_ complexes, the NBO E^(2)^ values are 2.5 and 7.0 kcal/mol for **10** and **15** (with *E*_orb_ values of –2.7 and –3.8 kcal·mol^–1^, respectively), while for the BBr_3_ complexes **11** and **16**, they are 2.8 and 7.7 kcal·mol^–1^ (with *E*_orb_ values of −3.2 and −4.4 kcal·mol^–1^).

The numerical differences between these two approaches stem from their distinct theoretical frameworks. EDA *E*_orb_ is a variational term that accounts for the total energy gain from the relaxation of all molecular orbitals upon complexation, including global polarization and all possible charge transfer pathways across the entire system. Conversely, NBO E^(2)^ uses a perturbative approach to isolate the stabilization arising specifically from a discrete, localized donor–acceptor pair, in this case the metal d_z_2 to the boron p_z_ or π* orbital. In these systems, the NBO *E* ^(2)^ values for the BH_3_ complexes are notably higher than the corresponding EDA *E*_orb_ values because NBO isolates the specific dative component of the interaction, whereas the EDA *E*_orb_ is an integrated term that reflects the net balance of orbital relaxation and can be influenced by the energy cost of electronic reorganization. Despite these quantitative variations, both methods are qualitatively consistent in confirming the superior donor ability of Pt over Pd and the transition from a typical noncovalent triel bond to an interaction with significant dative covalent character in the BH_3_ systems.

### 3.5 CSD Analysis

**FIGURE 7:**
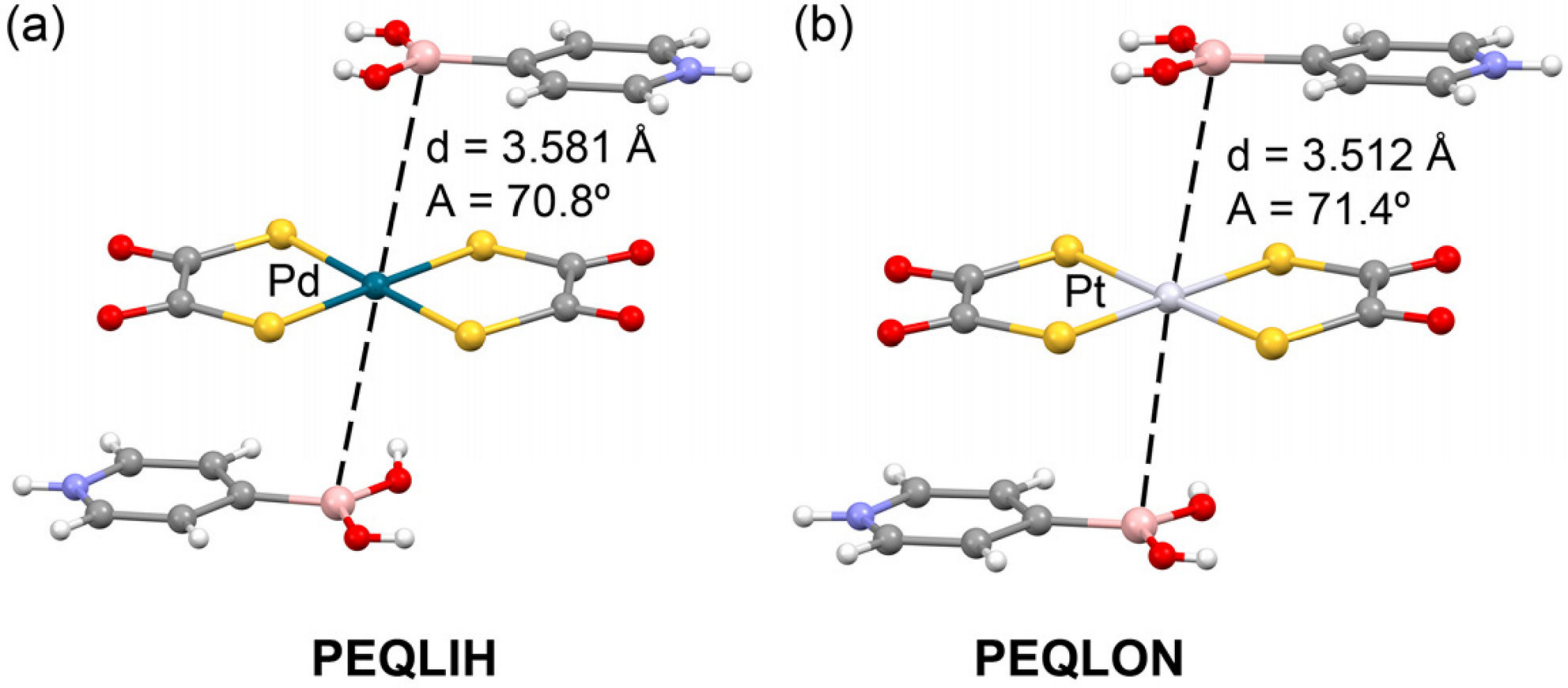
CSD structures of bis((4‐pyridinio)boronic acid) bis(dithiooxalato‐S, Sʹ)‐M(II) salts for M = Pd (PEQLIH), (a) and M = Pt (PEQLON), (b). Interaction distances (*d*, Å) and angles (*A*, °) are indicated, highlighting the M···B triel bond contact.

To provide experimental support for the existence of metal‐involving triel bonds, we conducted a search of the Cambridge Structural Database (CSD). We specifically investigated the occurrence of cocrystals or salts involving square‐planar complexes of Pd(II) and Pt(II) that exhibit M···B contacts. The search yielded two relevant hits, which are illustrated in Figure 7: the bis((4‐pyridinio)boronic acid) bis(dithiooxalato‐S, Sʹ)‐M(II) salts where M = Pd (PEQLIH) and M = Pt (PEQLON). Although the number of hits is currently very small, their identification provides crucial experimental evidence that this interaction exists in the solid state. Interestingly, these two hits are isostructural, differing only in the nature of the metal center. This allowed for a direct experimental comparison between Pd and Pt centers that parallels our theoretical findings. The observed M···B distances (3.581 Å for Pd and 3.512 Å for Pt) are comparable to the equilibrium distances (*d*) obtained for the halogenated BX_3_ series in our computational study. Furthermore, the interaction is shorter and more directional for the Pt complex (PEQLON, *d* = 3.512 Å, *A* = 71.4°) than for the Pd complex (PEQLIH, *d* = 3.581 Å, A = 70.8°), which is in excellent agreement with the theoretical results establishing Pt as a superior nucleophile.

## 4 Concluding Remarks

In conclusion, we have established the existence and nature of triel bonds formed between square planar Pd(II) and Pt(II) complexes and various boron‐based acceptors. Our multipronged theoretical approach reveals that while these interactions are moderately strong and highly directional, their underlying physical nature varies significantly across different donor families. The EDA results were instrumental in distinguishing these behaviors: the BH_3_ series is characterized by a dominant orbital component and partial covalency, whereas the BX_3_ and BAr_3_ series are primarily stabilized by electrostatic and dispersion forces, respectively. NBO analysis further confirmed these trends, identifying a significant charge transfer from the metal d_z_2 orbital into the boron π‐hole. Crucially, the analysis of the CSD provided experimental validation, showing that these interactions are present in known crystal structures with geometric features that mirror our computational predictions. By defining the triel bond as a viable member of the metal‐involving noncovalent interaction repertoire, this work provides a new framework for rational design in crystal engineering and supramolecular chemistry.

## Funding

This study was supported by Ministerio de Ciencia, Innovación y Universidades (PID2023‐148453NB‐I00).

## Conflicts of Interest

The authors declare no conflicts of interest.

## Supporting information

Figure S1 with the Kohn‐Sham (KS) molecular orbitals of a representative set of complexes. The cartesian coordinates of the optimized structures are provided.

## Data Availability

The data that supports the findings of this study are available in the supplementary material of this article.
