# Supplementary material for: Expansion of the Metal‐Involving Noncovalent Interaction Repertoire: The Case of Pd(II) and Pt(II) Triel Bonding
Source: Chemphyschem. 2026 Jul 7;27(13):e70485. doi: 10.1002/cphc.70485 (PMC13341402; doi:10.1002/cphc.70485)
Supplement: Supplementary file 1 — Figure S1 with the Kohn‐Sham (KS) molecular orbitals of a representative set of complexes. The cartesian coordinates of the optimized structures are provided. [file CPHC-27-e70485-s001.pdf]

Electronic supplementary information

for manuscript

Expansion of the Metal-Involving Noncovalent Interaction Repertoire: The Case of Pd(II) and Pt(II) Triel Bonding

By Rosa M. Gomila and Antonio Frontera

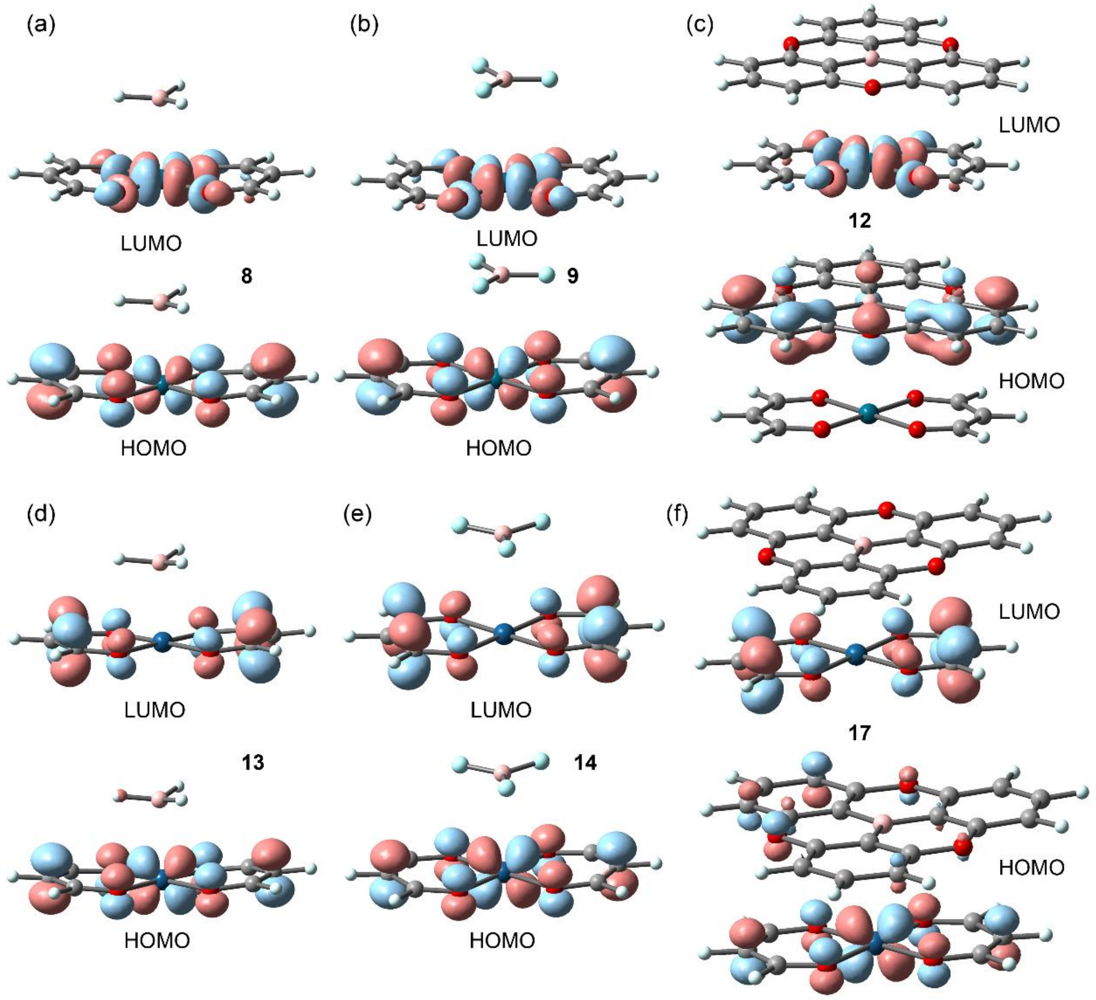

Figure S1. Kohn-Sham HOMO and LUMO plots for a representative set of complexes, computed at the PBE0-D3/def2-TZVP level of theory. Panels (a) and (b) show the frontier molecular orbitals of the Pd·BH_3 (8) and Pd·BF_3 (9) complexes. Panels (c) shows the Pd complex with the aromatic boron derivative (12). Panels (d) and (e) show the corresponding Pt·BH_3 (13) and Pt·BF_3 (14) complexes, respectively, and panel (f) shows the Pt complex with the aromatic boron derivative (17). The isosurface value is 0.04 au. Blue and red lobes indicate opposite phases of the wavefunction.
